# Dynamics of *Plasmodium vivax* populations in border areas of the Greater Mekong sub-region during malaria elimination

**DOI:** 10.1186/s12936-020-03221-9

**Published:** 2020-04-08

**Authors:** Yuling Li, Yubing Hu, Yan Zhao, Qinghui Wang, Huguette Gaelle Ngassa Mbenda, Veerayuth Kittichai, Saranath Lawpoolsri, Jetsumon Sattabongkot, Lynette Menezes, Xiaoming Liu, Liwang Cui, Yaming Cao

**Affiliations:** 1 Department of Immunology, College of Basic Medical Sciences, China Medical University, Shenyang, 110122 Liaoning China; 2 Emergency Department, The First Affiliated Hospital of Dalian Medical University, Dalian, 116011 Liaoning China; 3 Division of Infectious Diseases and International Medicine, Department of Internal Medicine, Morsani College of Medicine, University of South Florida, Tampa, FL 33612 USA; 4 Mahidol Vivax Research Unit, Faculty of Tropical Medicine, Mahidol University, Bangkok, Thailand; 5 Center for Global Health and Infectious Disease Research, College of Public Health, University of South Florida, Tampa, FL 33612 USA

**Keywords:** Malaria, *Plasmodium vivax*, Epidemiology, Population genetics, Microsatellites, Greater Mekong sub-region

## Abstract

**Background:**

Countries within the Greater Mekong Sub-region (GMS) of Southeast Asia have committed to eliminating malaria by 2030. Although the malaria situation has greatly improved, malaria transmission remains at international border regions. In some areas, *Plasmodium vivax* has become the predominant parasite. To gain a better understanding of transmission dynamics, knowledge on the changes of *P. vivax* populations after the scale-up of control interventions will guide more effective targeted control efforts.

**Methods:**

This study investigated genetic diversity and population structures in 206 *P. vivax* clinical samples collected at two time points in two international border areas: the China-Myanmar border (CMB) (n = 50 in 2004 and n = 52 in 2016) and Thailand-Myanmar border (TMB) (n = 50 in 2012 and n = 54 in 2015). Parasites were genotyped using 10 microsatellite markers.

**Results:**

Despite intensified control efforts, genetic diversity remained high (*H*_E_ = 0.66–0.86) and was not significantly different among the four populations (*P* > 0.05). Specifically, *H*_E_ slightly decreased from 0.76 in 2004 to 0.66 in 2016 at the CMB and increased from 0.80 in 2012 to 0.86 in 2015 at the TMB. The proportions of polyclonal infections varied significantly among the four populations (*P* < 0.05), and showed substantial decreases from 48.0% in 2004 to 23.7 at the CMB and from 40.0% in 2012 to 30.7% in 2015 at the TMB, with corresponding decreases in the multiplicity of infection. Consistent with the continuous decline of malaria incidence in the GMS over time, there were also increases in multilocus linkage disequilibrium, suggesting more fragmented and increasingly inbred parasite populations. There were considerable genetic differentiation and sub-division among the four tested populations. Temporal genetic differentiation was observed at each site (*F*_ST_ = 0.081 at the CMB and *F*_ST_ = 0.133 at the TMB). Various degrees of clustering were evident between the older parasite samples collected in 2004 at the CMB and the 2016 CMB and 2012 TMB populations, suggesting some of these parasites had shared ancestry. In contrast, the 2015 TMB population was genetically distinctive, which may reflect a process of population replacement. Whereas the effective population size (*N*_*e*_) at the CMB showed a decrease from 4979 in 2004 to 3052 in 2016 with the infinite allele model, the *N*_*e*_ at the TMB experienced an increase from 6289 to 10,259.

**Conclusions:**

With enhanced control efforts on malaria, *P. vivax* at the TMB and CMB showed considerable spatial and temporal differentiation, but the presence of large *P. vivax* reservoirs still sustained genetic diversity and transmission. These findings provide new insights into *P. vivax* transmission dynamics and population structure in these border areas of the GMS. Coordinated and integrated control efforts on both sides of international borders are essential to reach the goal of regional malaria elimination.

## Background

As the global incidence of malaria has been greatly reduced in recent years, *Plasmodium vivax* has become the main source of malaria infections in many endemic areas where *Plasmodium falciparum* and *P. vivax* co-exist [1]. This is also true for most areas of the Greater Mekong Sub-region (GMS) in Southeast Asia [2], which is actively pursuing regional malaria elimination. This shift in species dominance in the face of intensified control efforts highlights the remarkable adaptive potential and relative resilience of *P. vivax* to control measures [3]. In recognizing the challenge for eliminating vivax malaria, the GMS countries planned to eliminate falciparum malaria by 2025 and all malaria by 2030 [4]. As the malaria elimination plan progresses in the GMS, malaria displayed increasing heterogeneity in distribution, with transmission being concentrated along international borders [5]. These international borders are porous with intensive human migration, which poses a major threat of parasite introduction [6–8]. In addition, border regions have drastically different ecology, vector systems, human populations, and are subject to influences of wars and civil unrest; thus malaria transmission in border regions are often unstable [9]. While malaria surveillance has been strengthened in the GMS countries, it can be complemented by population genetic studies to define parasite diversity, population structure and migration among geographically separated transmission hotspots [10].

Since a major mechanism of generating genetic diversity in the malaria parasites is meiotic recombination of parasite strains in mosquito vectors, genetic diversity is intrinsically linked to the transmission intensity [11]. Likewise, it is expected that reduction of parasite population in response to intensified interventions will lead to reduced parasite genetic diversity. However, *P. vivax* seems to defy this principle. In most of its geographic range, *P. vivax* showed high levels of genetic diversity as revealed by population genetic studies using various genotyping markers [12–21]. Paradoxically, even in many low transmission settings, *P. vivax* still maintains high levels of diversity [22–26]. As a result, in areas of *P. vivax* and *P. falciparum* co-endemicity, these two parasites tend to exhibit markedly different genetic diversity and population structures, with *P. vivax* showing more stable transmission patterns [14, 15, 27–29]. Newly introduced parasites in areas where malaria had been eliminated may initially display low-level genetic diversity or even clonality [30, 31], but the parasites could rapidly re-establish diverse populations [32]. As a result, the shrinking *P. vivax* populations in areas with intensified control frequently showed relatively high genetic diversity, but substantial levels of multilocus linkage disequilibrium (LD) and population substructure [15, 17, 23, 33].

The scale-up of malaria control efforts in countries of the GMS has led to substantial changes in malaria epidemiology with a noticeable rising proportion of vivax malaria [5, 34]. In the China-Myanmar border (CMB) areas, *P. vivax* not only has become the predominant *Plasmodium* species in recent years [35], but also has caused several malaria outbreaks [9, 36]. Even in the international border regions, malaria transmission is concentrated in separated hotspots, and among which gene flow is expected to be low [37]. Final attacks to eliminate these hotspots, guided by accurate surveillance, will ensure the success of the regional elimination programme. An improved understanding of the transmission dynamics and the adaptive responses of the parasites to the control measures will also be essential to guide and monitor the progress of the elimination campaign. Recent studies of *P. vivax* populations from international border regions of Thailand with microsatellite (MS) markers clearly detected parasite population division, consistent with the separation of these parasite populations by the malaria-free central region [13, 38]. Here *P. vivax* clinical samples collected at different time points of the same study sites were used to determine the effect of intensified control efforts on genetic diversity and population dynamics of *P. vivax* in two border regions of the GMS.

## Methods

### Ethics statement

Written informed consent was obtained from all *P. vivax* patients or their legal guardians for participants under the age of 18 years. This study was approved by the Institutional Review Boards of Department of Disease Control, Thailand Ministry of Health and the Pennsylvania State University. Use of the de-identified blood samples was approved by the Ethics Review Committee of China Medical University.

### Study sites and *Plasmodium vivax* clinical samples

In order to investigate the change of the *P. vivax* populations in response to intensified malaria control efforts in the GMS, *P. vivax* isolates were collected at two time points from two different areas of the CMB and Thailand-Myanmar border (TMB) (Additional file 1: Fig. S1). The CMB area has been traditionally malaria hyper-endemic with both *P. falciparum* and *P. vivax* transmission. Although recent malaria control efforts have sharply reduced the *P. falciparum* incidence [35], *P. vivax* incidence remained consistently high and even experienced outbreaks in recent years [9, 36]. At the CMB, 50 samples were collected in 2004 to represent the parasite population prior to the implementation of malaria elimination measures, while 52 samples collected in 2016 represented the parasites after scaling-up of malaria control. These samples were all collected from symptomatic *P. vivax* patients at the hospital and clinics in the adjacent Laiza Township (Myanmar) and Nabang Township (China). These hospital and clinics are located within 2 km from each other, and are thus considered as a single study site. In Thailand, Tak Province has been among the most malaria-endemic areas. In the past decade, malaria incidence in Tak has experienced more than 10-fold reduction, from 13,706 in 2012 to 1364 in 2016 [13]. In 2012 and 2016, *P. vivax* accounted for 57.5% and 86.7% of the total cases, respectively (http://203.157.41.215/malariar10/index_newversion.php). Fifty *P. vivax* clinical samples obtained in 2012 from the Tha Song Yang hospital, Tak Province represented the parasites before the endorsement of malaria elimination plan, and were used to compare with samples collected from the same hospital in 2015 [13]. While the CMB 2004 samples were collected as part of routine surveillance, the rest of the samples were collected from passive case detection implemented in these study sites for the International Center of Excellence for Malaria Research (ICEMR) project. Finger-prick blood samples were collected from symptomatic patients after obtaining informed consent. Malaria diagnosis was based on microscopy of Giemsa-stained thin and thick smears. For confirmed *P. vivax* cases, ~ 100 μl of finger-prick blood were spotted onto Whatman filter paper, dried and stored in individually zipped plastic bags.

### Microsatellite genotyping

Parasite genomic DNA was isolated from the dried blood spots on filter paper according to the protocol of QIAmp DNA Mini kit (Qiagen, Hilden, Germany). The final purified DNA was eluted into 35 μl of elution buffer and used immediately or stored at − 20 °C until further use.

A volume of 2 μl of purified DNA was used as the template for malaria parasite detection by a genus-specific and species-specific nested PCR targeting the *Plasmodium* 18S rRNA genes [39]. Ten MS markers (MS1, MS2, MS5, MS6, MS7, MS9, MS10, MS12, MS15, MS20) previously used to differentiate *P. vivax* populations in Thailand [13] were used for genotyping these *P. vivax* samples. A multiplex primary PCR was done using all 10 primer pairs and followed by singleplex secondary PCR with a fluorescently labelled (6-FAM, VIC or NED) forward primer as previously described [40]. PCR products were used for GeneScan fragment analysis on an ABI3730xl capillary electrophoresis platform (Applied Biosystems) using the size standard LIZ500. Genotype calling was facilitated with GeneMapper Version 4.0. The predominant allele and any additional alleles with peak height at least one-third of the height of the predominant allele per locus were scored [41]. Genotyping success was defined as the presence of at least one allele at a given locus in a given sample.

### Data analysis

Isolates containing more than one peak for any marker were considered to be multiple clone infections. The multiplicity of infection (MOI) was defined as the maximum number of alleles observed at any of the loci investigated. The mean MOI was calculated from the individual samples for each study site. Alleles were binned using the TANDEM software [42]. Isolates with one allele at all markers, were considered monoclonal infections. An infection was defined as polyclonal if more than one allele was observed at one or more loci. For isolates with more than one allele at any of the loci, the alleles with the highest peak were used to construct the dominant haplotypes as previously described [14]. Input files for the various population genetics software programs were created using CONVERT version 1.31.

### Population diversity and differentiation

The indices of genetic diversity within populations, such as the number of polymorphic loci, the number of haplotypes (Nh), the number of alleles (Na), the mean allelic richness, and the expected heterozygosity (*H*_E_) were calculated using Arlequin version 3.11 [43] and GenAlEx version 6.5 [44]. In order to assess the genetic differentiation among populations, pairwise comparisons were measured by calculating *F*_ST_ using GenAlEx version 6.5. To estimate the partitioning of genetic variance for different hypothesized population groupings, analysis of molecular variance (AMOVA) was performed using GenAlEx version 6.5.

### Linkage disequilibrium (LD)

Multilocus LD in each population was calculated using the program LIAN 3.7 [45] with 50,000 iterations for burn-in and then 100,000 Markov Chain Monte Carlo (MCMC) iterations. Samples with missing data were excluded for this analysis. To avoid detecting false inbreeding resulting from clonal propagation and physical linkage, this analysis was performed for the combined dataset of single and dominant haplotypes and for unique haplotypes only. In addition, LD was analysed using only monoclonal and low-complexity (containing just one multi-allelic locus) samples. Since MS2 and MS5 both localize to chromosome 6 and MS12 and MS15 to chromosome 5, MS2 and MS12 were excluded in separate analysis as they had higher levels of missing data.

### Effective population size (*N*_e_)

The effective population size was calculated using the stepwise mutation model (SMM) and infinite alleles model (IAM) as previously described [11]. Mutation rates for *P. vivax* are lacking and thus the *P. falciparum* mutation rate of 1.59 × 10^−4^ (95% confidence interval: 6.98 × 10^−5^–3.7 × 10^−4^) was used [46]. In addition, the mutation rate of *P. vivax* calibrated from an eradicated European strain (5.57 × 10^−7^), which was ~ 3500× lower than that estimated for *P. falciparum*, was also used [47].

### Bottleneck analysis

A heterozygosity excess test at the population level was used to detect the recent population bottleneck using BOTTLENECK 1.2.02 [48] with the SMM [49] and the two-phase model (TPM) [50]. The Wilcoxon signed rank test, a robust statistic for testing less than 20 polymorphic loci, was executed in the model in order to ascertain the probability of significant heterozygote excess. Since the method implemented in the bottleneck has low power [51] unless the decline is greater than 90%, the Garza-Williamson index was computed using Arlequin version 3.11. The Garza-Williamson index is the mean ratio of the number of alleles at a given locus to the range in allele size, i.e., M = (k/r), where k is the number of alleles and r is the allelic range (i.e., the difference in repeat units between the shortest and the longest alleles at a locus) [52]. This measure is based on the assumption that in a bottleneck event, the number of alleles decreases faster than the allelic range because the latter is only reduced if the shortest and/or longest allele is lost, whereas the loss of any allele reduces the former. For the Garza-Williamson index, M < 0.68 indicates a bottleneck, whereas M > 0.80 indicates no reduction of effective population size.

### Population structure

Deviations from Hardy–Weinberg equilibrium could indicate the presence of population structure or inbreeding [53]. To investigate whether haplotypes cluster into distinct genetic populations (K) among the defined geographic areas, a Bayesian analysis of population structure was conducted using STRUCTURE version 2.3.2 [54]. The admixture model was used and the posterior probability of the grouping number (K = 1–10) was estimated by the MCMC method with 10 separate runs to evaluate the consistency of the results. Each run was estimated as 10,000 steps with 100,000-step burn-in. The best-fit number of grouping was evaluated using ΔK in the STRUCTURE HARVESTER version 0.6.93 tool [55, 56]. The CLUMPP version 1.1.2 [57] and DISTRUCT 1.1 [58] were used to display the partitioning clusters.

To visualize genetic relationships among the parasite isolates from the four populations, an individual-based principal coordinate analysis (PCoA) was conducted in GenAlEx version 6.5 using the genetic distances among MS genotypes. In addition, phylogenetic relationships amongst *P. vivax* isolates were analysed using the Neighbour-Joining method [59] implemented in MEGA7 [60]. Minimum spanning tree of parasite genotypes constructed by the goeBURST algorithm using the Phyloviz software v1.1 (http://www.phyloviz.net/).

## Results

### Within-host and population diversity

A total of 206 *P. vivax* isolates from two areas of the GMS were genotyped at 10 MS markers. Of the 10 MS markers, failure rates for MS7 (16.5%) and MS9 (15.0%) were the top two highest. Complete genotyping data at all 10 loci were obtained for 171 (83.0%) isolates. Since the maximum number of missing MS data for each sample was 4, all samples were included in the analysis. The 10 MS markers all were polymorphic with each one having 2-20 alleles when all parasite populations were considered and allele frequencies varied among the four populations (Additional file 2: Table S1).

Overall, 72 (34.9%) parasite isolates contained polyclonal infections (more than one peak for at least one MS marker), and the proportions of multiclonal infections were significantly different among the four parasite populations (*P *< 0.05, Pearson Chi square test) (Table 1). At both sites, along with the reduction in malaria incidence, there were substantial decreases in the proportion of multiclonal infections (*P* < 0.05, Pearson Chi square test). At the CMB, compared with the 48.0% polyclonal infections observed in 2004, this proportion decreased to 30.7% in 2016. Similarly, at the TMB, the percentage of polyclonal infections also experienced a considerable decrease from 40.0% in 2012 to 23.7% in 2016. Accordingly, this was reflected in the reduction of mean MOI from 1.48 in 2004 to 1.33 in 2016 at the CMB, and from 1.50 in 2012 to 1.36 in 2015 in the TMB area (Table 1).

**Table 1:** Genetic diversity of *P. vivax* populations at the China–Myanmar border (CMB) and Thailand–Myanmar border (TMB)

Populations (N)	Nh	*H*_*E*_± SE	Na ± SE	Mean allelic richness	Multiclonal infections (%)^#^	MOI
CMB2004 (50)	42	0.76 ± 0.04	10.80 ± 1.20	12.20	48.0	1.48
CMB2016 (52)	34	0.66 ± 0.08	9.30 ± 1.40	8.80	30.7	1.33
TMB2012 (50)	39	0.80 ± 0.03	11.40 ± 1.10	11.20	40.0	1.50
TMB2015 (54)	42	0.86 ± 0.02	13.7 ± 1.04	12.80	23.7	1.36

*Nh* number of haplotypes, *H*_*E*_ expected heterozygosity, *SE* standard error, *Na* number of alleles, *MOI* multiplicity of infection

^#^Significant difference among the four populations (*P* < 0.05, Pearson Chi square test)

Despite the overall reduction in MOI, the genetic diversity of parasite populations at both sites remained high (Table 1). First, allele richness in these parasite populations remained high (Table 1). At the CMB, allele richness in parasite populations showed a substantial reduction from 12.2 in 2004 to 8.8 in 2016. In contrast, the allele richness in parasite populations at the TMB did not show much change, at 11.2 in 2012 and 12.8 in 2015. Second, haplotype diversity was high; a total of 162 haplotypes were observed and no haplotypes were shared among the four populations. The 2004 CMB and 2015 TMB parasite populations harboured the greatest number of haplotypes (Table 1). A slight decrease in the expected heterozygosity was observed at the CMB from 0.76 in 2004 to 0.66 in 2016, although this did not reach statistical significance (*P* > 0.05). In comparison, the genetic diversity of parasite populations at the TMB had a slight increase from 0.80 in 2012 to 0.86 in 2015 (Table 1). This high genetic diversity may reflect substantially large parasite populations sustaining continued transmission at these two border areas. With both the SMM and IAM models, the effective population size *N*_*e*_ at the CMB was moderate and showed a ~ twofold decrease over the past decade, whereas *N*_*e*_ in western Thailand remained high and even had a ~ twofold increase in recent years (Table 2). These effective population sizes were much larger when the new *P. vivax* mutation rate was used (Additional file 3: Table S2).

**Table 2:** Effective population sizes (*N*_*e*_) of the *P. vivax* populations at the China–Myanmar border (CMB) and Thailand–Myanmar border (TMB)

Populations (N)	SMM	95% CI	IAM	95% CI
CMB2004 (50)	12,862	5527–29,299	4979	2139–11,341
CMB2016 (52)	6014	2584–13,700	3052	1311–6952
TMB2012 (50)	18,867	8108–42,979	6289	2702–14,326
TMB2015 (54)	43,724	18,790–99,601	10,259	4408–23,368

The *N*_e_ was estimated using two models, the stepwise mutational model (SMM) and the infinite allele model (IAM) with the *P. falciparum* mutation rate

*CI* confidence interval

### Multilocus LD and population bottlenecks

The standardized index of association I^S^A used to measure the degree of inbreeding revealed significant multilocus LD in the *P. vivax* populations from the both CMB (*P* < 0.0001) and TMB (*P* < 0.0001) (Table 3). Multilocus LD was also retained when only unique haplotypes or monoclonal and low-complexity samples were considered or only one MS marker per chromosome (excluding MS2 and MS12) was analysed (Additional file 4: Table S3), confirming that LD was not the result of false reconstruction or physical linkage of MS markers. Also noteworthy is that the LD patterns barely changed when unique haplotypes were used for analysis, suggesting that there is no evidence of epidemic-like transmission patterns in any of the sites/time points. Considering temporal changes in multilocus LD, there was an increasing trend of LD in parasite populations at the CMB from 2004 (I^S^A = 0.0236) to 2016 (I^S^A = 0.0329) and at the TMB from 2012 (I^S^A = 0.0366) to 2015 (I^S^A = 0.0586) (Table 3), which may contribute to the potential effect of population reduction in both sites with the scale-up of malaria control measures.

**Table 3:** Multilocus linkage disequilibrium (*I*^*S*^*A*) in the *P. vivax* populations analysed using all 10 microsatellite loci

Population (N)	All haplotypes			Unique haplotypes			Monoclonal haplotypes		
	n	*I*^*S*^*A*	p	n	*I*^*S*^*A*	p	n	*I*^*S*^*A*	p
CMB2004 (50)	44	0.0320	< 0.00001	42	0.0279	0.00123	24	0.0322	0.0167
CMB2016 (52)	34	0.0492	0.00002	34	0.0492	0.00002	23	0.0583	0.00029
TMB2012 (50)	39	0.0442	< 0.00001	39	0.0442	< 0.00001	21	0.0199	0.0431
TMB2015 (54)	49	0.0679	< 0.00001	42	0.0199	0.00784	40	0.0137	0.00762

*n* number of haplotypes used in the analysis, *I*^*S*^*A* standardized index of association

To detect whether there were significant changes in the parasite population size, BOTTLENECK analysis was performed under SMM and TPM, and the Garza–Williamson index was computed (Table 4). The SMM detected significant deficiency in *H*_*E*_ from the mutation-drift equilibrium, indicating events of population size reduction with possible clonal expansion in all four populations. The TPM only detected such events in the TMB populations. Likewise, the mean Garza-Williamson index was less than 0.68 in all the four populations, also suggesting a reduction of the effective population size.

**Table 4:** Bottleneck analysis using the stepwise mutation model (SMM), two-phase model (TPM) and the Garza–Williamson statistic

Populations (N)	SMM			TPM			G–W statistic
	Excess-H_E_	Deficient-H_E_	2-tails	Excess-H_E_	Deficient-H_E_	2-tails	(Mean ± SD)
CMB2004 (50)	0.991	0.01221*	0.0244*	0.61523	0.42285	0.84570	0.14 ± 0.13
CMB2016 (52)	0.997	0.00342*	0.0068*	0.58984	0.45508	0.91016	0.14 ± 0.09
TMB2012 (50)	0.996	0.00488*	0.0097*	0.00049*	1.00000	0.00098*	0.17 ± 0.13
TMB2015 (54)	0.995	0.00684*	0.0137*	0.00684*	0.99512	0.01367*	0.17 ± 0.12

*G–W* Garza–Williamson index, *SD* standard deviation

* For both excess-H_E_ and deficient-H_E_, P-values were from one-tailed analysis and * indicates significance at *P* < 0.05

### Genetic differentiation and population structure

Genetic differentiation of the *P. vivax* populations in the GMS were evaluated by estimating the Wright’s fixation index *F*_ST_. As expected, the contemporary *P. vivax* populations from the CMB and TMB showed considerable genetic differentiation (*F*_ST_ = 0.169 and 0.237). The CMB *P. vivax* populations showed moderate differentiation between 2004 and 2016 (*F*_ST_ = 0.081), whereas the TMB parasite populations had substantial differentiation between 2012 and 2015 (*F*_ST_ = 0.133, Table 5). Interestingly, the 2004 CMB parasite population also showed little genetic differentiation from those collected from the 2012 TMB population (*F*_ST_ = 0.064), but significant genetic differentiation from the 2015 TMB population (*F*_ST_ = 0.172). Overall, AMOVA indicated 87% of the variations was attributed to variations within populations, and 13% among populations. Specifically, at the CMB, 93% of the variations was attributed to variations within populations and 7% among populations, whereas at the TMB, 88% of the variation was attributed to variations within populations and 12% among populations.

**Table 5:** Pairwise comparison of *F*_ST_ among *P. vivax* populations from the China–Myanmar border (CMB) and Thailand–Myanmar border (TMB)

Populations	CMB2004	CMB2016	TMB2012
CMB2016	0.081*		
TMB2012	0.064*	0.169*	
TMB2015	0.172*	0.237*	0.133*

*P values obtained after permutation test at *P* < 0.01

**Fig. 1:**
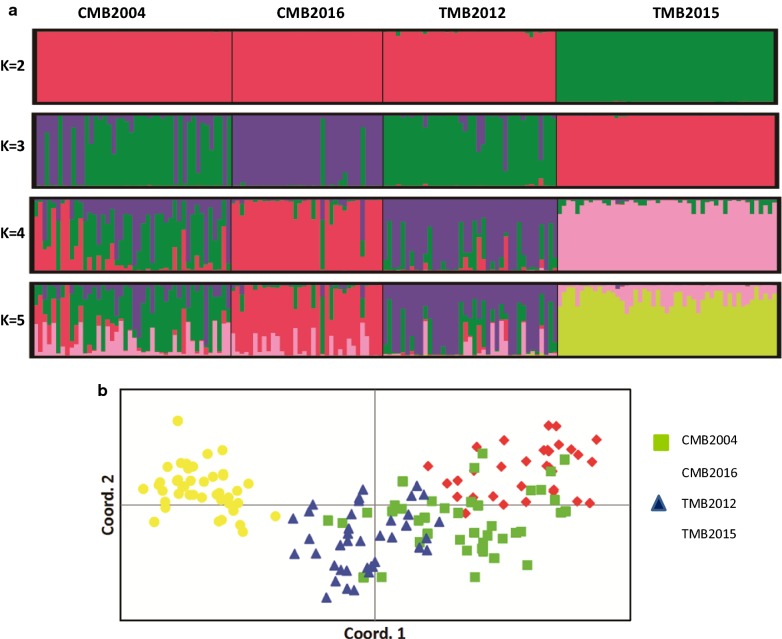
**a** Population genetic structure of *Plasmodium vivax* from four populations from the GMS (K = 2–5). The analysis assigns *P. vivax* haplotypes to a defined number of genetic clusters (K) based on genetic distance. Vertical bars indicate individual *P. vivax* haplotype and colours represent the ancestry co-efficient (membership) within each cluster. **b** Principal coordinate analysis of *P. vivax* haplotypes from four parasite populations. Colours indicate the geographic origin of each sample. *CMB* China–Myanmar border, *TMB* Thailand–Myanmar border

STRUCTURE analysis showed a clear distribution pattern of parasite haplotypes and demonstrated multiple sub-populations (Fig. 1a). Most parasites from the TMB collected in 2015 were separated from all other populations. Using the delta K method, the most likely number of sub-populations was identified as 2 (Additional file 5: Fig. S2). At K = 2, populations collected from the TMB in 2015 formed a separate cluster from the rest of the populations. At K = 3, in addition to the TMB2015 cluster, the CMB 2016 samples also formed a cluster, with admixture in samples from the CMB 2004 and TMB 2012. At K = 4, admixing between the CMB 2004 and the TMB 2012 parasite populations became more evident (Fig. 1a), which corroborated the weak genetic differentiation between these two populations from *F*_ST_ analysis (*F*_ST_ = 0.064, Table 5). At K = 5, the CMB 2004 and TMB 2012 parasites were substantially admixed (Fig. 1a), suggesting similar ancestry for these two parasite populations.

**Fig. 2:**
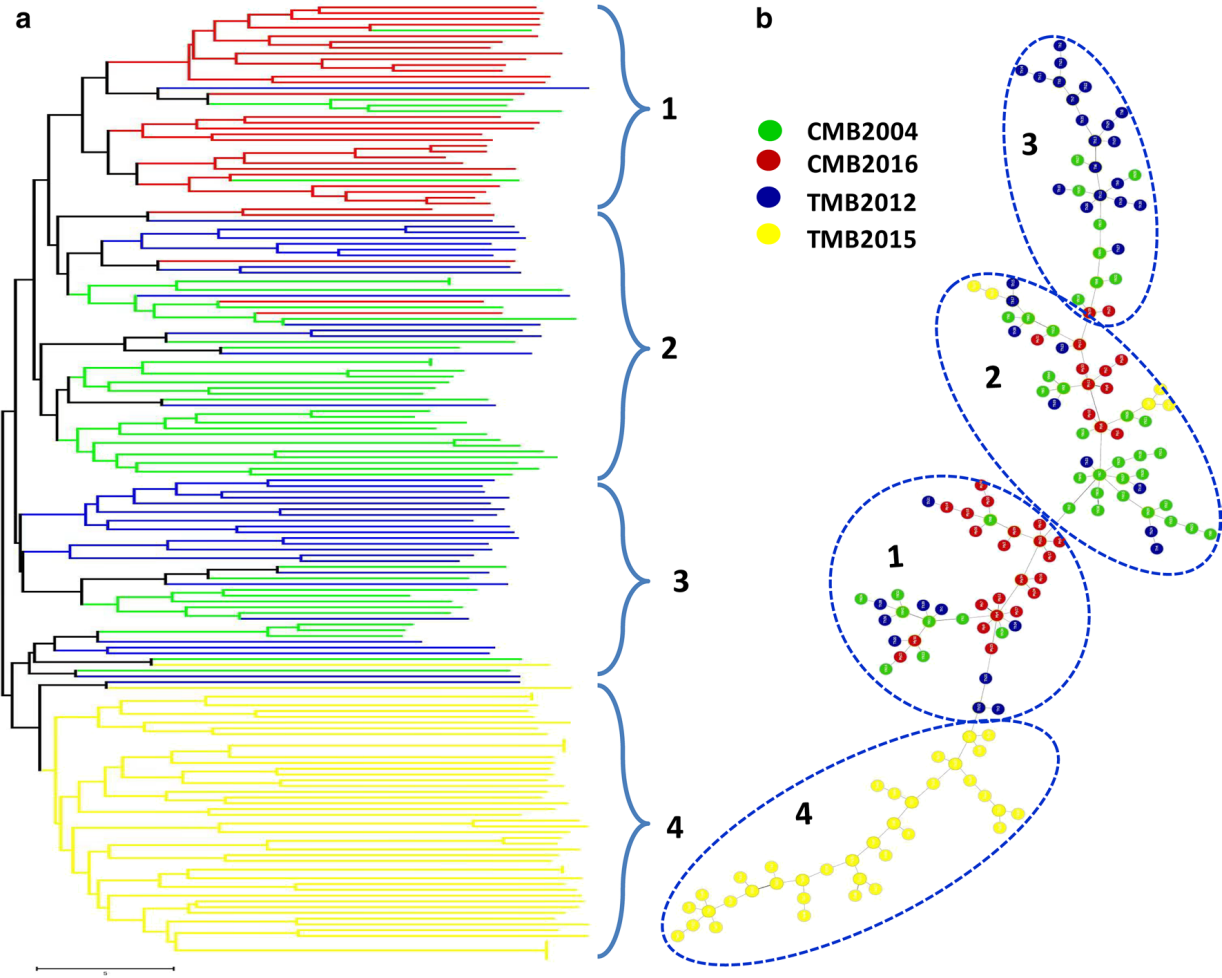
**a** Phylogenetic analysis of *Plasmodium vivax* isolates from the four parasite populations. Relatedness amongst haplotypes was defined by calculating the pairwise distance and visualized by drawing an unrooted phylogenetic tree using a Neighbour-Joining method. **b** Minimum spanning tree of parasite genotypes with each circle representing a haplotype. Colours indicate the geographic origin of each sample. *CMB* China–Myanmar border, *TMB* Thailand–Myanmar border

In line with the STRUCTURE analysis, PCoA confirmed the genetic separation of the four parasite populations. PC1 and PC2 explained 7.37 and 5.16% of the variances, respectively, and grouped the parasites into two main clusters. The three more contemporary samples TMB 2012, TMB 2015, and CMB 2016 formed individual clusters with no overlap, whereas the older CMB 2004 parasite population showed haplotype admixture with the TMB 2012 and CMB 2016 (Fig. 1b). The phylogenetic tree and network analysis further corroborated the findings of substantial regional population structure and local clustering of haplotypes (Fig. 2).

## Discussion

With *P. vivax* becoming dominant in most parts of the GMS, a better understanding of transmission dynamics and population structure of this parasite is crucial to guide targeted control efforts and to achieve the goal of malaria elimination under the changing settings of malaria epidemiology [61]. This work aimed to study the changes of parasite populations in the GMS during the elimination process, with samples collected in two international border areas before and after the scale-up of malaria control interventions. This study demonstrated that despite intensified control efforts in the GMS, there were only moderate levels of reduction in parasite genetic diversity and the proportion of polyclonal infections. Meanwhile the *P. vivax* parasite displayed significant LD and substantial genetic differentiation. The possible epidemiological processes driving these patterns of diversity and population structure are discussed below.

The GMS is malaria hypo-endemic with malaria transmission occurring along international borders. In both CMB and TMB, camps for refugees or internally displayed people have been established, where there is constant human population movement. Genetic diversity of *P. vivax* populations in the two border regions of the GMS was moderately high with the expected heterozygosity ranging from 0.66 to 0.86. Compared to previous studies which used a nearly identical set of MS markers, the *H*_*E*_ values in the current study were remarkably close to those from low-to-moderate transmission areas in Asia, such as Vietnam (0.68), Thailand (0.76), central China (0.816), Laos (0.75), and India (0.72), as well as South America, such as Colombia (0.79) and Brazil (0.71) [22, 29, 30, 62, 63]. Despite strengthened malaria control efforts in both regions, *H*_*E*_ only decreased slightly at the CMB and remained relatively unchanged in the TMB. This may reflect the differences in the time span of samples collected at the two sites: 12 years at the CMB versus 2–3 years at the TMB. An earlier study conducted using the CMB 2011–2013 samples also revealed stable genetic diversity (*H*_*E*_ = 0.82) in these parasite populations [16]. The high genetic diversity in low transmission areas is a common phenomenon that has been reported in numerous *P. vivax*-endemic areas [22–24, 28]. Reasons for this are not completely clear but are considered to be multifactorial [64]. Of direct relevance to the border settings is the mobility of the camp and border populations along the borders, which could introduce new parasites and enhance genetic diversity of the parasite populations. The earlier study conducted at the CMB provided evidence on the existence of extensive gene flow among border communities and across international borders [16]. Unlike that international borders are present as a potential barrier for the spread of *P. falciparum* strains in the GMS [65], landscape factors at the CMB do not appear to impede gene flow [16]. Cross-border frequent human migration facilitates the importation of malaria into low-transmission areas and this represents a major challenge to malaria elimination in the GMS [66].

Regardless of the time span difference, both the CMB and TMB populations showed substantial reduction (17–18%) in the proportion of polyclonal infections and MOI. Yet, the proportion of polyclonal infections was unchanged at the CMB from 29% in 2011–2013 [16] to 30.7% in 2016. Compared to the hypo-endemic settings in central China (5.9%) and Malaysia (17.6–29.2%) and hyper-endemic settings such as Papua New Guinea (63.8–88.3%), Cambodia (89.6%), Indonesia (23–79%), and Ethiopia (35%) [15, 22, 40, 67–69], the proportions of polyclonal infections (30.7% and 23.7%) in the CMB and TMB parasite samples from recent years remained moderately high. In addition, parasite populations in these regions remained relatively large, especially at the TMB, although the estimates from the new *P. vivax* mutation rate seemed not realistic. Multiclonal infections, together with the large population size, will increase the chance of recombination, resulting in the observed high genetic diversity. It is therefore important to further enhance local malaria control efforts to achieve substantial reduction of the parasite populations in these transmission hotspots.

Another noticeable change is the detection of significant multilocus LD in the parasite populations despite high genetic diversity. This again seems to be a common finding in many vivax low-endemicity settings such as Indonesia, Papua New Guinea, South Korea, India, Vietnam, Colombia, and Brazil [15, 32, 62, 64, 70, 71]. Significant LD against a background of high diversity may reflect the existence of multiple spatially clustered infections within a defined population, which might have arisen from rapid reduction in transmission and effective population size as malaria control interventions have intensified in the GMS. The overall trend of increasing LD in both sites and elsewhere in Thailand [13] is a demonstration of reduced overall parasite genetic complexity during malaria elimination.

Across different border regions of Thailand, a previous MS analysis detected substantial parasite population differentiation [13]. This study also detected considerable divergence of the parasite populations across time and space. Phylogenetic and network analyses showed that most of the parasites from individual populations fell into distinct clades or clusters, indicating that the parasite populations in the GMS have become increasingly differentiated. The high malaria transmission areas along the international border regions of the GMS were largely connected a decade ago [72]. Consistently, the older parasite population from the CMB 2004 showed remarkable degrees of clustering with the CMB 2016 and TMB 2012 samples, which may reflect shared ancestry of these parasites in the recent past. In contrast, the current malaria map illustrated the presence of separated pockets of transmission foci from intensified control efforts of the malaria elimination campaign, which limit gene flow within the GMS [73]. The genetic differentiation between the contemporary parasite populations is an indication of independent evolution of these isolated parasite populations as also documented for the *P. falciparum* parasites [37]. In addition, heterogeneity within vector populations could affect transmission [74] and population structure of the parasites [75]. The notable shift in vector populations observed in western Thailand may also be responsible for shaping the parasite population structure [76]. Moreover, the parasite populations studied here have undergone apparent population bottlenecks, suggesting population reduction, or limited vectors or human movement within the defined geographic areas. At the CMB, the epidemic malaria transmission documented in the camps for internally displaced people and surrounding villages in 2013 and the detected reduction of effective population size may result from expansion of certain parasite isolates such as those that are resistant to the frontline treatment [77, 78]. Interestingly, the TMB 2015 samples were quite genetically distinct, and even differed considerably from the 2012 parasite from the same region, which may suggest population replacement. This is plausible as the border parasite populations are subject to extensive parasite introduction [6], and introduced parasites are capable of re-establishing a more diverse population within a short period [32]. In addition, new malaria interventions such as mass drug administration [79] may have accelerated the parasite population changes. The establishment of community-based malaria posts equipped with rapid diagnostic tests and antimalarial drug in the eastern Myanmar has led to a sharp decline of Myanmar malaria patients seeking medical service at the Thai side [80], which may also be related to the parasite population change.

## Conclusions

Overall, this investigation demonstrated that the scale-up of malaria control interventions for malaria elimination in the GMS has resulted in both spatial and temporal *P. vivax* population differentiation. However, the persistently high genetic diversity, moderate levels of polyclonal infections, and considerably large effective population sizes demand concerted efforts of border nations to implement more effective measures targeting this resilient parasite. Especially in western Thailand, both parasite genetic diversity and effective population size have experienced slight increases in recent years. The large degree of population movement, rapid ecological changes and complex vector population dynamics in western Thailand are very conducive to sustained malaria transmission [81]. With a large proportion of infection linked to cross-border travel, it will be important to strengthen malaria control measures on both sides of the border. At these low-endemicity settings, the identification of the source of residual infections and implementation of targeted control may be critical for the final phase of elimination [82, 83]. These findings provide novel insights for malaria surveillance and enhanced control efforts especially targeting the fragmented populations to reach the goal of malaria elimination by 2030.

## Supplementary information

**Additional file 1: Fig. S1.** Map of GMS showing the two border regions (stars) where *Plasmodium vivax* clinical samples were collected.

**Additional file 2: Table S1.** The number of alleles (#) and allelic richness of each microsatellite marker in the four populations.

**Additional file 3: Table S2.** Effective population size (*N*_*e*_) of the *P. vivax* populations estimated using the SMM and IAM models.

**Additional file 4: Table S3.** Multilocus linkage disequilibrium (*I*^*S*^*A*) in the *P. vivax* populations analysed using 8 microsatellite loci.

**Additional file 5: Fig. S2.** Estimation of the optimal number of populations (K) using the deltaK/K method.

## Data Availability

The datasets supporting the conclusions of this article are available in additional files.

## Abbreviations

AMOVA: Analysis of molecular variance
CI: Confidence interval
CMB: China–Myanmar border
*F*_ST_: Wright’s fixation index
GMS: Greater Mekong sub-region
*H*_*E*_: Expected heterozygosity
IAM: Infinite alleles model
I^S^A: Standardized index of association
LD: Linkage disequilibrium
MOI: Multiplicity of infection
Na: The number of alleles
*N*_*e*_: The effective population size
Nh: Number of haplotypes
PCoA: Principal coordinate analysis
SD: Standard deviation
SMM: Stepwise mutation model
TMB: Thailand–Myanmar border
TPM: Two-phase model
