# Supplementary material for: Dynamics of Plasmodium vivax populations in border areas of the Greater Mekong sub-region during malaria elimination
Source: Malar J. 2020 Apr 8;19:145. doi: 10.1186/s12936-020-03221-9 (PMC7140319; doi:10.1186/s12936-020-03221-9)
Supplement: Supplementary file 1 — Additional file 1: Fig. S1. Map of GMS showing the two border regions (stars) where Plasmodium vivax clinical samples were collected. [file 12936_2020_3221_MOESM1_ESM.pptx]

## Slide 1
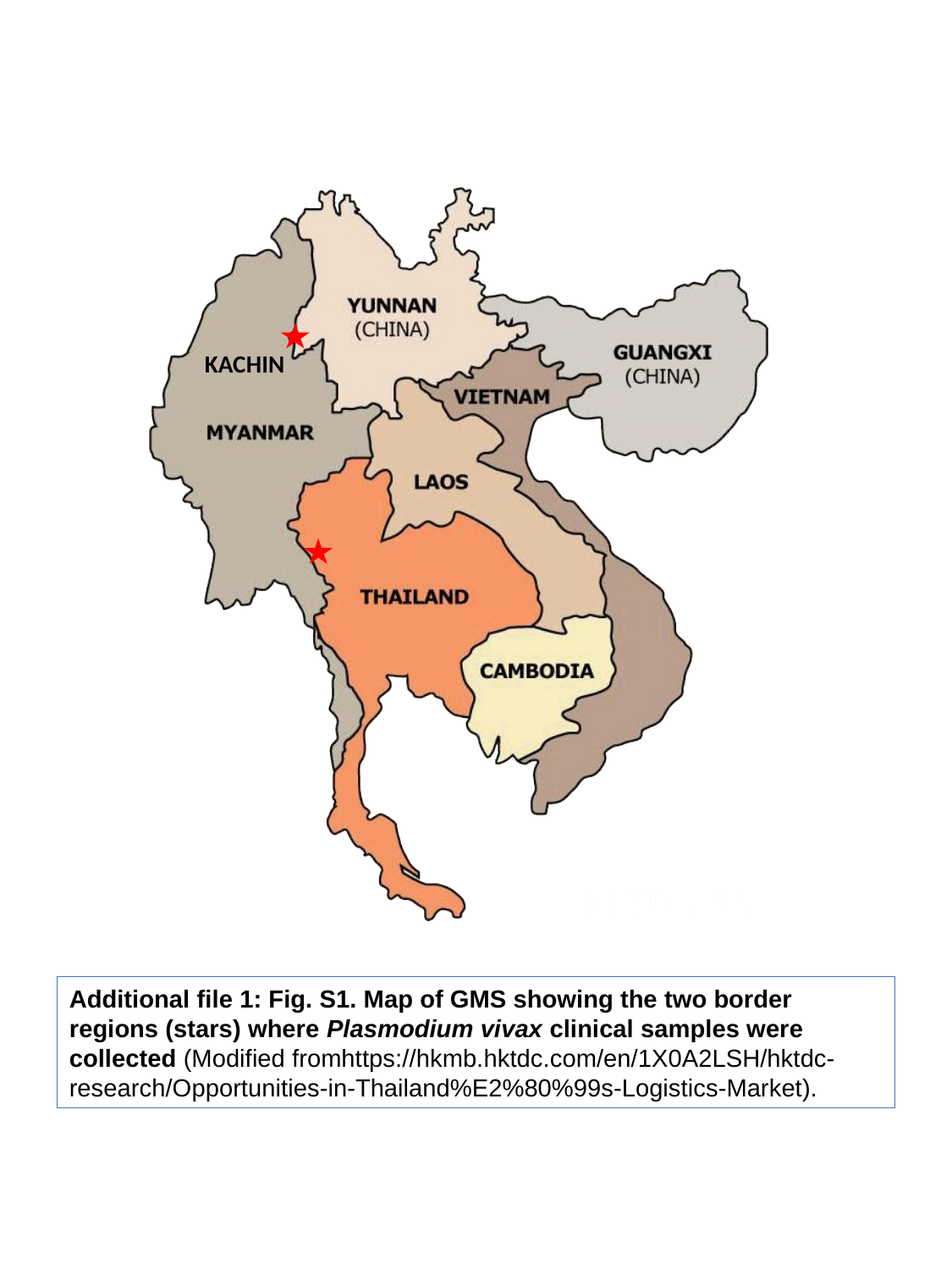

KACHIN
Additional file 1: Fig. S1. Map of GMS showing the two border regions (stars) where Plasmodium vivax clinical samples were collected (Modified fromhttps://hkmb.hktdc.com/en/1X0A2LSH/hktdc-research/Opportunities-in-Thailand%E2%80%99s-Logistics-Market).
