# Supplementary material for: Dynamics of Plasmodium vivax populations in border areas of the Greater Mekong sub-region during malaria elimination
Source: Malar J. 2020 Apr 8;19:145. doi: 10.1186/s12936-020-03221-9 (PMC7140319; doi:10.1186/s12936-020-03221-9)
Supplement: Supplementary file 2 — Additional file 2: Table S1. The number of alleles (#) and allelic richness of each microsatellite marker in the four populations. [file 12936_2020_3221_MOESM2_ESM.docx]

**Additional file 2: Table S1. The number of alleles (#) and allelic richness of each microsatellite marker in the four populations.**

| **MS Markers** | **CMB2004** | | **CMB2016** | | **TMB2012** | | **TMB2015** | |
| --- | --- | --- | --- | --- | --- | --- | --- | --- |
|  | # | Richness | # | Richness | # | Richness | # | Richness |
| MS1 | 9 | 8.540 | 6 | 5.649 | 9 | 8.449 | 6 | 5.673 |
| MS2 | 20 | 18.689 | 10 | 9.471 | 16 | 15.740 | 18 | 16.700 |
| MS5 | 9 | 8.660 | 6 | 5.419 | 10 | 9.576 | 13 | 12.708 |
| MS6 | 6 | 5.924 | 8 | 7.414 | 7 | 6.801 | 17 | 16.017 |
| MS7 | 11 | 10.539 | 16 | 16.000 | 11 | 10.940 | 15 | 14.066 |
| MS9 | 13 | 12.601 | 15 | 14.911 | 12 | 11.940 | 12 | 10.891 |
| MS10 | 10 | 9.858 | 9 | 8.543 | 12 | 11.475 | 13 | 11.803 |
| MS12 | 9 | 8.468 | 12 | 10.517 | 7 | 6.479 | 16 | 15.275 |
| MS15 | 13 | 12.534 | 9 | 8.527 | 12 | 11.367 | 11 | 10.104 |
| MS20 | 8 | 7.148 | 2 | 1.654 | 18 | 16.014 | 16 | 14.824 |

CMB, China-Myanmar border; TMB, Thailand-Myanmar border.
