# Supplementary material for: Dynamics of Plasmodium vivax populations in border areas of the Greater Mekong sub-region during malaria elimination
Source: Malar J. 2020 Apr 8;19:145. doi: 10.1186/s12936-020-03221-9 (PMC7140319; doi:10.1186/s12936-020-03221-9)
Supplement: Supplementary file 3 — Additional file 3: Table S2. Effective population size (Ne) of the P. vivax populations estimated using the SMM and IAM models. [file 12936_2020_3221_MOESM3_ESM.docx]

Additional file 3: Table S2. Effective population size (*N_e_*) of the *P. vivax* populations estimated using the SMM and IAM models.

| **Populations** | **SMM** | **95%CI** | **IAM** | **95%CI** |
| --- | --- | --- | --- | --- |
| CMB2004 (n=50) | \| 3,671,703 \| \| --- \| | 1,929,376–74,368,686 | 1,421,304 | 746,855–28,787,878 |
| CMB2016 (n=52) | 1,716,902 | 902,183–34,775,086 | 871,264 | 457,824–17,647,058 |
| TMB2012 (n=50) | 5,385,996 | 2,830,188–109,090,909 | 1,795,332 | 943,396–36,363,636 |
| TMB2015 (n=54) | 11,225,405 | 5,898,633–227,365,491 | 2,757,117 | 1,448,787–55,844,155 |

*N_e_* was estimated using the *P. vivax* mutation rate of 5.57×10^–7^ (95% CI 2.75×10^–8^–1.06×10^–6^). SMM, stepwise mutational model; IAM, infinite allele model.

95% CI, 95% confidence interval.

Table 3. Multilocus linkage disequilibrium (*I^S^A*) for all loci .

| Population | All haplotypes, all loci | | | Unique haplotypes, all loci | | | Monoclonal haplotypes, all loci | | |
| --- | --- | --- | --- | --- | --- | --- | --- | --- | --- |
|  | n | *I^S^A* | p | n | *I^S^A* | p | n | *I^S^A* | p |
| CMB2004 | 44 | 0.0320 | <0.00001 | 42 | 0.0279 | 0.00123 | 24 | 0.0322 | 0.0167 |
| CMB2016 | 34 | 0.0492 | 0.00002 | 34 | 0.0492 | 0.00002 | 23 | 0.0583 | 0.00029 |
| TMB2012 | 39 | 0.0442 | <0.00001 | 39 | 0.0442 | <0.00001 | 21 | 0.0199 | 0.0431 |
| TMB2015 | 49 | 0.0679 | <0.00001 | 42 | 0.0199 | 0.00784 | 40 | 0.0137 | 0.00762 |

n, number of haplotypes used in the analysis; *I^S^A,* standardized index of association.

Multilocus linkage disequilibrium (*I^S^A*) for one locus per chromosome^#^

| Population | All haplotypes, 8 loci | | | Unique haplotypes, 8 loci | | | Monoclonal haplotypes, 8 loci | | |
| --- | --- | --- | --- | --- | --- | --- | --- | --- | --- |
|  | n | *I^S^A* | p | n | *I^S^A* | p | n | *I^S^A* | p |
| CMB2004 | 44 | 0.0236 | 0.0174 | 42 | 0.0181 | 0.0352 | 24 | 0.0067 | 0.0342 |
| CMB2016 | 34 | 0.0329 | 0.0056 | 34 | 0.0329 | 0.0056 | 23 | 0.0399 | 0.0174 |
| TMB2012 | 39 | 0.0366 | 0.00004 | 39 | 0.0366 | 0.00004 | 21 | 0.0048 | 0.034 |
| TMB2015 | 49 | 0.0586 | <0.00001 | 42 | 0.0194 | 0.00454 | 40 | 0.0192 | 0.00512 |

n, number of haplotypes used in the analysis; *I^S^A,* standardized index of association.

# MS2 and MS12 were excluded from analysis.

Table 4. Bottleneck analysis

| Populations | SMM | | | TPM | | | G–W statistic |
| --- | --- | --- | --- | --- | --- | --- | --- |
|  | Excess–H_E_ | Deficient–H_E_ | 2–tails | Excess–H_E_ | Deficient–H_E_ | 2–tails | Mean ± SD |
| CMB2004(n=50) | 0.991 | 0.01221* | 0.0244* | 0.61523 | 0.42285 | 0.84570 | 0.14±0.13 |
| CMB2016(n=52) | 0.997 | 0.00342* | 0.0068* | 0.58984 | 0.45508 | 0.91016 | 0.14±0.09 |
| TMB2012(n=50) | 0.996 | 0.00488* | 0.0097* | 0.00049* | 1.00000 | 0.00098* | 0.17±0.13 |
| TMB2015(n=54) | 0.995 | 0.00684* | 0.0137* | 0.00684* | 0.99512 | 0.01367* | 0.17±0.12 |

* For both Excess–H_E_ and Deficient–H_E_, P–values were from one–tailed analysis and * indicates significance at P < 0.05.

G–W, Garza–Williamson index; SD, standard deviation.

Table 5. Pairwise comparison of F_ST_ among *P. vivax* populations from China–Myanmar border and Thai– Myanmar border

| Population | CMB2004 | CMB2016 | Thailand2012 |
| --- | --- | --- | --- |
| CMB2016 | 0.081* |  |  |
| TMB2012 | 0.064* | 0.169* |  |
| TMB2015 | 0.172* | 0.237* | 0.133* |

*P values obtained after permutation test at P < 0.01.
