# Supplementary material for: Dynamics of Plasmodium vivax populations in border areas of the Greater Mekong sub-region during malaria elimination
Source: Malar J. 2020 Apr 8;19:145. doi: 10.1186/s12936-020-03221-9 (PMC7140319; doi:10.1186/s12936-020-03221-9)
Supplement: Supplementary file 4 — Additional file 4: Table S3. Multilocus linkage disequilibrium (ISA) in the P. vivax populations analysed using 8 microsatellite loci. [file 12936_2020_3221_MOESM4_ESM.docx]

**Additional file 4: Table S3.** **Multilocus linkage disequilibrium (*I^S^A*) in the *P. vivax* populations analysed using 8 microsatellite loci ^#^**

| **Population (N)** | **All haplotypes** | | | **Unique haplotypes** | | | **Monoclonal haplotypes** | | |
| --- | --- | --- | --- | --- | --- | --- | --- | --- | --- |
|  | n | *I^S^A* | p | n | *I^S^A* | p | n | *I^S^A* | p |
| CMB2004 (50) | 44 | 0.0236 | 0.0174 | 42 | 0.0181 | 0.0352 | 24 | 0.0067 | 0.0342 |
| CMB2016 (52) | 34 | 0.0329 | 0.0056 | 34 | 0.0329 | 0.0056 | 23 | 0.0399 | 0.0174 |
| TMB2012 (50) | 39 | 0.0366 | 0.00004 | 39 | 0.0366 | 0.00004 | 21 | 0.0048 | 0.034 |
| TMB2015 (54) | 49 | 0.0586 | <0.00001 | 42 | 0.0194 | 0.00454 | 40 | 0.0192 | 0.00512 |

n, number of haplotypes used in the analysis; *I^S^A,* standardized index of association.

# MS2 and MS12 were excluded from analysis.
