# Supplementary material for: Dynamics of Plasmodium vivax populations in border areas of the Greater Mekong sub-region during malaria elimination
Source: Malar J. 2020 Apr 8;19:145. doi: 10.1186/s12936-020-03221-9 (PMC7140319; doi:10.1186/s12936-020-03221-9)
Supplement: Supplementary file 5 — Additional file 5: Fig. S2. Estimation of the optimal number of populations (K) using the deltaK/K method. [file 12936_2020_3221_MOESM5_ESM.pptx]

## Slide 1
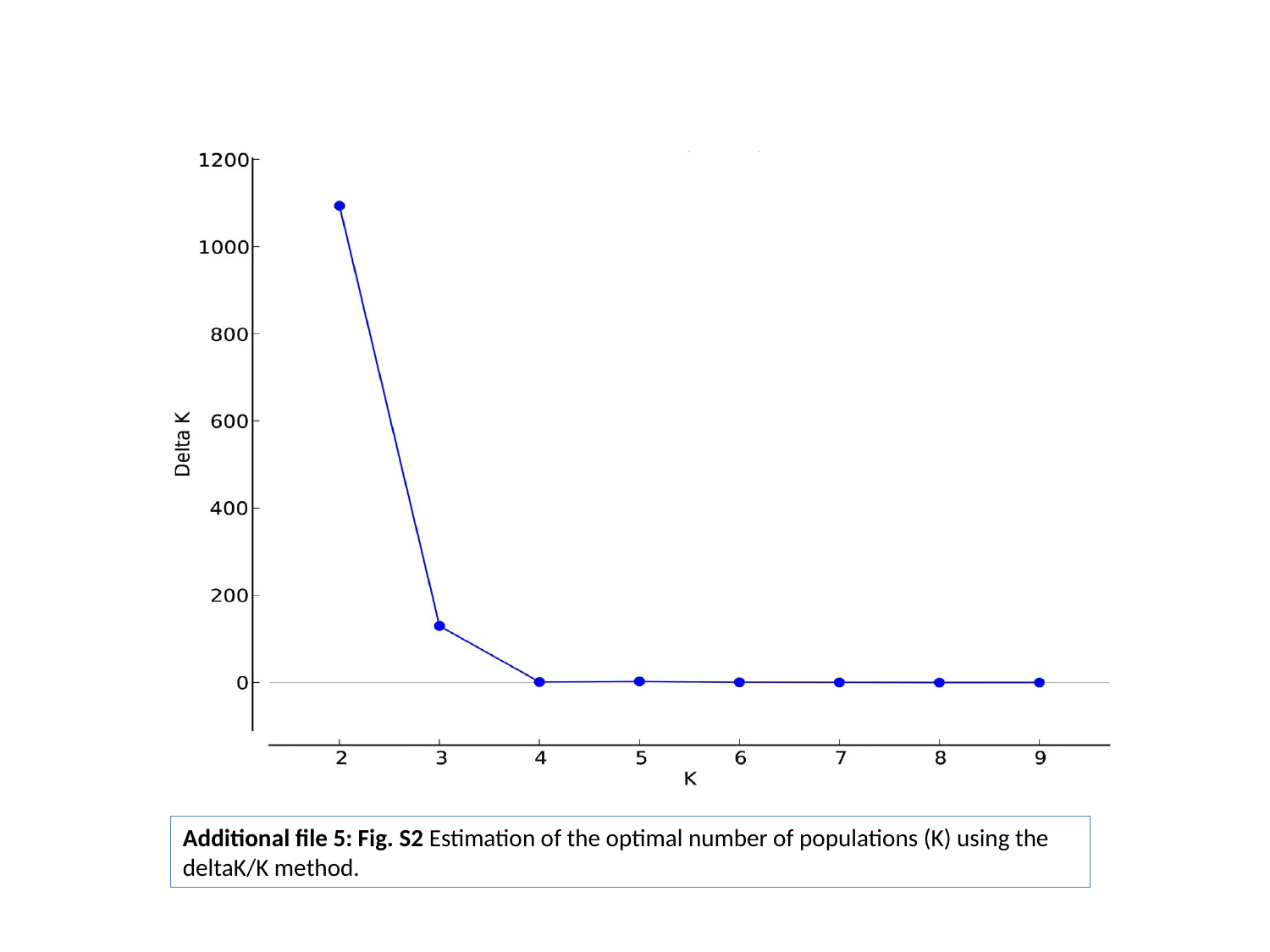

Additional file 5: Fig. S2 Estimation of the optimal number of populations (K) using the deltaK/K method.
